# A System for the Synchronized Recording of Sonomyography, Electromyography and Joint Angle

**DOI:** 10.2174/1874120700701010077

**Published:** 2007-12-11

**Authors:** Q.H Huang, Y.P Zheng, X Chena, J.F He, J Shi

**Affiliations:** a Department of Health Technology and Informatics, The Hong Kong Polytechnic University, Kowloon, Hong Kong SAR, China; b Department of Electronic Engineering, City University of Hong Kong, Kowloon, Hong Kong SAR, China

**Keywords:** Muscle, fatigue, ultrasound, electromyography, EMG, sonomyography, SMG.

## Abstract

Ultrasound and electromyography (EMG) are two of the most commonly used diagnostic tools for the assessment of muscles. Recently, many studies reported the simultaneous collection of EMG signals and ultrasound images, which were normally amplified and digitized by different devices. However, there is lack of a systematic method to synchronize them and no study has reported the effects of ultrasound gel to the EMG signal collection during the simultaneous data collection. In this paper, we introduced a new method to synchronize ultrasound B-scan images, EMG signals, joint angles and other related signals (e.g. force and velocity signals) in real-time. The B-mode ultrasound images were simultaneously captured by the PC together with the surface EMG (SEMG) and the joint angle signal. The deformations of the forearm muscles induced by wrist motions were extracted from a sequence of ultrasound images, named as Sonomyography (SMG). Preliminary experiments demonstrated that the proposed method could reliably collect the synchronized ultrasound images, SEMG signals and joint angle signals in real-time. In addition, the effect of ultrasound gel on the SEMG signals when the EMG electrodes were close to the ultrasound probe was studied. It was found that the SEMG signals were not significantly affected by the amount of the ultrasound gel. The system is being used for the study of contractions of various muscles as well as the muscle fatigue.

## 1 INTRODUCTION

Electromyography (EMG) is the most commonly used approach for the assessment of skeletal muscles in both clinics and research areas. Ultrasound is also attracting increased interests in this field in recent years. They have been successfully applied to study the changes of muscle functions and activities [1-5]. Ultrasound imaging has been widely used in both static and dynamical evaluation of the dimensional properties of muscle and detection of muscle lesions [1, 4-8], due to its advantages of being portable, noninvasive, non-ionizing, inexpensive, and easy-to-use, in comparison with other imaging modalities. 2D ultrasound images can provide detailed anatomical information in cross-sectional planes inside the muscles and therefore enhance the assessment efficiency. Meanwhile, EMG has been playing an important role particularly in analyzing muscle physiological behaviors [3]. Normally, the amplitude of EMG can be used for characterizing muscle activities. In comparison with needle EMG, surface electromyography (SEMG) provides a relatively simple, non-invasive, and fairly specific way to assess the activation of superficial muscles, hence it has been widely used in ergonomics, biomechanics, sports science, and kinesiology.

Conventionally, ultrasound and EMG are employed independently in clinical practices. However, many applications, such as work-related musculoskeletal disorders [9] and evaluation of muscle strength [10], require the quantitative assessments of both muscle architectural changes and muscle fiber EMG activities, which can provide more useful evidences for studying muscle properties and diagnosing muscle-related diseases. Recently, more attention has been paid to studying muscles using both ultrasound and EMG. Hodges *et al*. [11] compared ultrasound measures of muscle architecture with EMG measures of the activities of specific muscles during isometric contractions and concluded that ultrasound imaging could be used to detect muscle architecture changes and activities. McMeeken *et al*. [12] investigated the relationship between EMG activity and transversus abdominis thickness measured by real-time ultrasound during various levels of maximum voluntary contraction (MVC). Ferreira *et al*. [13] used both ultrasonography and EMG to study the changes of abdominal muscle activities in people with low back pain (LBP). And the fatigue of muscles and tendons was investigated using both SEMG and ultrasound imaging [14,15]. We have also used sonomyography (SMG), which is defined as real-time change of muscle thickness detected using ultrasound during muscle contraction, to assess muscle fatigue [16]. Using a well established relation between muscle dimensional changes and EMG readings, the muscle activities during a specific action can be better investigated. The functions and properties for different muscles can be assessed more comprehensively. Thus, it is important to simultaneously collect the two signals for studying their relationship. These studies mentioned above tried to collect ultrasound and EMG signals at the same time thus the information from the two signals could be correlated to each other at the same time point. Although some of these studies used a trigger signal to simultaneously acquire both the ultrasound and EMG signals, the two signals were actually independently collected without a specific synchronization procedure and the data analysis had to be performed off-line. We have earlier developed a system for the real-time data collection and analysis of EMG and ultrasound images during muscle contractions [16, 17]. However, since the time-lags between different data streams, which were collected by different devices, could not be accurately measured, it is not possible to investigate the time responses of different signals in relation to the muscle contraction.

Moreover, the trigger signal used in previous reports [11, 12, 13] was normally input between the EMG amplifier/ultrasound scanner and the computer. Hence, the time delay caused by both the EMG amplifier and the ultrasound imaging device could not be detected. This would help to investigate the temporal relationship between the EMG parameters and the muscle architectural changes more precisely. In addition, previous studies [9, 11] using both ultrasound imaging and SEMG signals were conducted by attaching the SEMG electrodes to the skin very close to the ultrasound transducer. However, whether the ultrasound coupling gel has any effect on the measurement of SEMG signals was not addressed in those studies. Due to the fact that ultrasound coupling gel is a sort of conductive material, the skin electrical impedance may be altered. And the more, the water content in the ultrasound gel may hydrate the stratum conerum layer to increase the conductivity of the skin and the EMG signal may also conduct through the ultrasound gel. Therefore, the impact of ultrasound gel on the collection of SEMG signals is worth to investigate.

Accordingly, in this study we developed a method to synchronize SMG, SEMG, and other signals, such as the joint angle data from an electronic goniometer and the torque signal from a dynamometer. A test was also conducted to study the effect of ultrasound coupling gel on the SEMG signals. The system development and experiment methods are described in section 2. The results obtained from this system are given in section 3. The potential applications and limitations of the system are discussed and conclusions are drawn in section 4.

## 2 MATERIALS AND METHODS

### 2.1 System Design

**Fig. (1):**
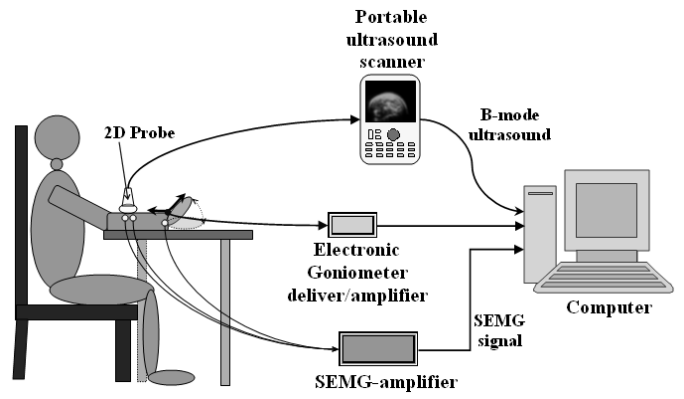
The diagram of the system for continuously and simultaneously capturing B-mode ultrasound, SEMG and joint angle signals.

As shown in (Fig. **1**), the system was comprised of a portable ultrasound scanner (SonoSite 180PLUS, SonoSite, Inc., Bothell, WA, USA), an electronic goniometer (XM110, Penny & Giles Biometrics, Inc., UK), a custom-developed EMG amplifier, and a PC with 2.8 GHz Pentium IV microprocessor and 512 MB RAM. The probe (L38/10-5 MHz, SonoSite, Inc., Bothell, WA, USA) of the ultrasound scanner was used to scan the muscle tissues of subjects. The video signal generated by the ultrasound scanner was digitized by a video capture card (NI-IMAQ PCI-1411, National Instruments Corporation, Austin, TX, USA) installed in the PC into a sequence of 2D images in real-time (25 Hz). The SEMG signals captured from the EMG bipolar Ag-Agcl electrodes (Axon Systems, Inc., NY, USA) attached on the skin were amplified by the custom-made EMG amplifier and filtered by a 10-300Hz band-pass analog filter. The filtered signal was digitized into 12 bits with a sampling rate of 4 KHz and 256 data points using a data acquisition card (NI-DAQ 6024E, National Instruments Corporation, Austin, TX, USA), which could continuously collect the SEMG signal and other related analog signals, such as joint angles measured by the electronic goniometer and torques generated by muscle contraction. The data were grouped into frames with each frame containing 256 points of EMG data and 1 ultrasound image.

**Fig. (2):**
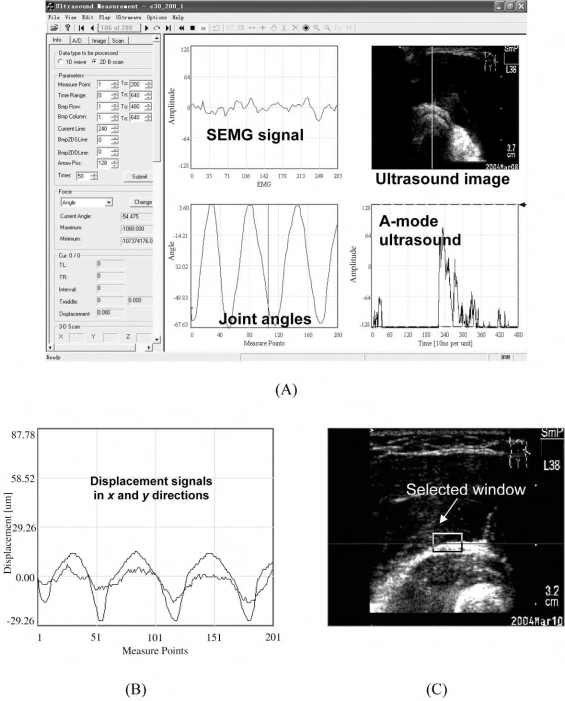
The interface of the multithreaded program for data acquisition and analysis. (**A**) The main window of the program for displaying ultrasound, SEMG, and angle; (**B**) The tracking window selected in the B-mode ultrasound images; and (**C**) The corresponding displacement curves.

A multithreaded program for ultrasound measurement of motion and elasticity (UMME,  http://www.tups.org/) was developed using Visual C++ to acquire the B-mode ultrasound and SEMG signals simultaneously (Fig. **2A**). The captured signals and images could be recorded and displayed in real-time during the data acquisition and could also be replayed off-line.

### 2.2. Synchronization of Acquisition

**Fig. (3):**
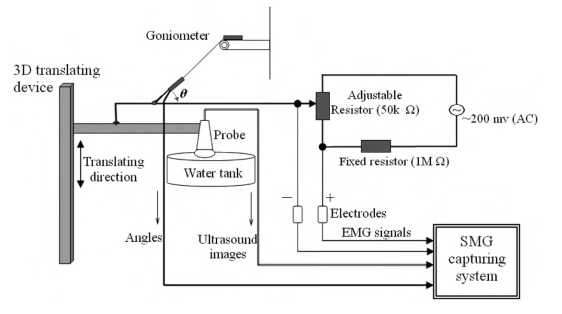
The diagram for the temporal calibration. When the ultrasound probe was moved by the 3D translating device, the angle *θ* and the mimicked EMG signal would also be changed.

**Fig. (4):**
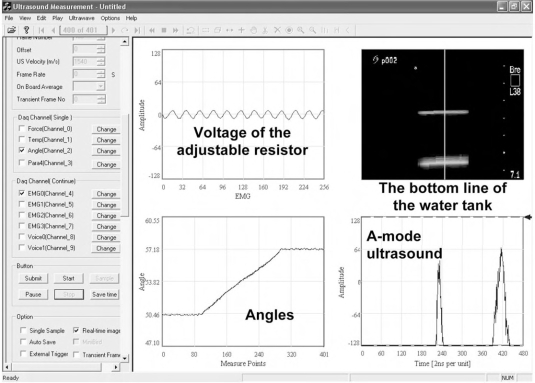
The captured images and signals for the temporal calibration shown in the program interface.

Because the original signals were processed using several electronic devices before transferred into the computer and a multithreaded program was used for data acquisition in the system, there tended to be a temporal shift between each pair of different data streams. In order to accurately analyze SEMG and muscle deformation signals, a temporal calibration procedure was necessary to be performed to synchronize these different signals. In this study, we proposed a temporal calibration system based on the approach reported in [18], as illustrated in (Fig. **3**). In the calibration system, a circuit was designed including a fixed resistor (100 MΩ) and a linearly adjustable resistor (60mm Slide potentiometer, ALPS Electric, Japan) and a signal generator (33120A, Hewlet-Packard Company, USA) was used to provide the circuit with a sinusoidal signal with an amplitude of 200 mV and a frequency of 100 Hz, which mimicked EMG signals and could be detected by the EMG amplifier. A 3D translating device (Parker Hannifin Corporation, Irvine, CA, USA) was used to control the up-and-down movement of the ultrasound probe immerged in a water tank. The change of the distance between the water tank bottom and the ultrasound probe was employed to mimic the muscle deformation during contraction, while the angle change sensed by the goniometer mimicked the change of the joint angle with one sensing part connected to the 3D translating device and the other fixed on a plate. As shown in (Fig. **3**), the movement of the 3D translating device caused the vertical translation of the ultrasound probe, which resulted in the positional translation of the bottom line of the water tank in the sequence of ultrasound images. The movement also caused the translation of the adjustable resistor’s knob that could change the input voltage of the EMG amplifier *via* two electrodes, leading to the change of the angle of the goniometer. The voltage change and the tangent of the angle *θ* (Fig. **3**) generated by the goniometer were linearly proportional to the translating distance of the ultrasound probe. Therefore, we could firstly capture these signals during the movement of the 3D translating device as illustrated in (Fig. ****4****), and then normalize the three different data streams. The optimal time offset between two different signals corresponded to the shifted time segment that made the two signals most correlated to each other. The temporal calibration experiments were repeated 5 times. The averaged results were used for the synchronization among the ultrasound image, SEMG RMS, and joint angle collected from the subject.

### 2.3 Signal and Image Processing

The muscle contraction involves the changes of architectures, which can be monitored using ultrasound in real-time. In order to detect the dimensional changes of the muscle, a 2D cross-correlation algorithm was used to track the shifts of a pair of selected image blocks in a sequence of B-mode frames [17]. The windows indicating tissue boundaries of interest were selected in the first frame of the ultrasound images collected before muscle contraction as a reference. The tracking algorithm was then applied to find the region most similar to the references in the subsequent image frames. This could be performed in real-time during the data collection and the displacement of the selected image blocks could be drawn as a signal in a window, as demonstrated in (Figs. **2B** and **2C**), allowing the operator to have a quick preview and evaluation of the experimental results. The ultrasound images could also be recorded together with other signals for off-line analysis.

The RMS of the amplitude for the captured SEMG signal was calculated to represent the EMG activities of muscles during the contraction. In addition to the analog filtering achieved by the EMG amplifier, the SEMG RMS signals were further filtered using a low-pass FIR filter with a cutoff frequency of 5 Hz and compared with the muscle deformation extracted from the ultrasound images. The correlations among SEMG activities, the muscle architectural changes and the joint angles might provide more comprehensive evaluation for muscle activities.

### 2.4 Subjects and Experiments

**Fig. (5):**
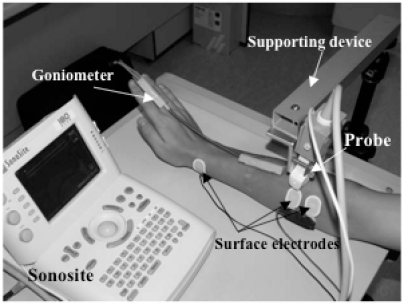
The experimental setup for collecting the ultrasound images, SEMG, and wrist angle.

Five healthy subjects (2 females and 3 males) with a mean (SD) age of 27.8 (3.4) years, a mean weight of 60.8 (7.7) kg, and a mean height of 1.67 (0.05) m, participated in the experiments. During the experiment, the subject was asked to put his/her right forearm on a flat table, as illustrated in (Fig. **5**). The ultrasound probe was fixed by a supporting device of a camera tripod to image the subject’s extensors and radius surfaces clearly. A pair of EMG electrodes was attached to the skin surface near the probe with an additional one attached to the right wrist for providing the reference electrical signal. All subjects were requested firstly to put their palms on the table, then to extend their wrists to push the palms backwards as much as possible, and finally put their palms on the table again to relax their wrist. This action was repeatedly performed guided by a metronome (MT-40, Wittner, Germany) with a beep rate of 30 beats per min. The goniometer was used to measure the wrist angles of the subjects, as shown in (Fig. **5**). The angle data were collected *via* the DAQ card and synchronized with ultrasound images and SEMG signals. In this study, the sampling rate of B-mode ultrasound was 14 Hz. For each experiment, the time for data acquisition was approximately 14 s and 200 frames of B-mode ultrasound images and SEMG signals were captured.

**Fig. (6):**
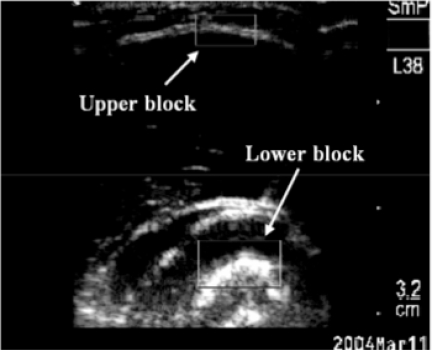
Two selected image blocks for tracking the muscle deformations.

As described earlier, the deformation of the extensor muscle was measured by tracking the distance between two selected windows that contained the upper and lower boundaries of the muscle, respectively (Fig. **6**). The measured tissue deformation, represented by the percentage change in comparison with the original muscle thickness when no action was made by the subjects, was correlated with the SEMG RMS and the angles read from the goniometer.

### 2.5 Tests of Ultrasound Coupling Gel

**Fig. (7):**
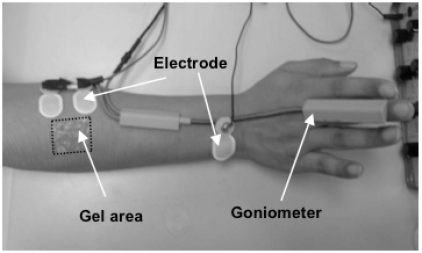
The experimental setup for the tests of the effects of the ultrasound gel on the SEMG collection. The gel areas tested were 0, 1, 6, 10 and 24 cm^2^ respectively.

Because the system was used to study the properties and activities of a particular muscle, the EMG electrodes should be placed near the ultrasound probe during experiments. However, it is still unclear whether the ultrasound coupling gel has any effect on the collection of the SEMG signals. Additional tests were conducted to investigate this problem in the current study. The right forearms of eight young healthy subjects (4 males and 4 females) with a mean (SD) age of 27.4 (4.1) years were tested. As illustrated in (Fig. **7**), a pair of EMG electrodes was attached close to the ultrasound gel area. The subjects were instructed to extend their wrists to keep the wrist angles at approximately 50 degrees for about 12 s in each test. For each subject, five tests were performed and the ultrasound gel area on the skin surface was 1×1, 2×3, 2×5, 3×8 cm^2^, respectively. The long axis was along the forearm. The center of the gel patch was fixed at the same location during the measurement. It was approximately at the location where ultrasound probe was placed during the simultaneous data collection of EMG and ultrasound images. The subjects rested for 5 min between two tests. The SEMG RMS of each test was calculated, and for the gel areas of 1, 6, 10 and 24 cm^2^ the testing results were further normalized by the following calculation

(1) RMS1→24=RMS1→24−RMS0/RMS0

where *RMS*_1→24_ is the result calculated from original SEMG signal for the tests with gel attached (gel area > 0 cm^2^), RMS1→24 is the normalized value for the *RMS*_1→24_, and RMS_0_ the averaged testing result without ultrasound gel (gel area = 0 cm^2^). With the results obtained, the effect of ultrasound gel was investigated using two-way ANOVA (SPSS, SPSS Inc., Chicago, IL, USA), and *p*<0.05 was considered to be statistically significant.

## 3 RESULTS

Using the joint angle as a reference signal, the time delay of ultrasound images was 106±12 ms (mean±SD), and that of SEMG RMS was 182±15 ms (mean±SD). Fig. (**8**) shows the typical transient signals of the measured muscle deformation, SEMG RMS, and wrist angle of one subject after the adjustment for the time delay. The preliminary application of the system and the image processing have demonstrated that the muscle deformation could be successfully obtained from the 2D ultrasound data and synchronized with other signals related to muscular activities. The results of other subjects showed similar patterns.

Fig. (**9**) shows the relationships between the SEMG RMS and joint angle signals and between the muscle deformation and joint angle signals, respectively. Obvious hysteresis phenomena were observed in the results of this typical subject, indicating there were phase shifts between the signals. The results of other subjects showed similar behaviors. It was found that second-order polynomial regressions could well describe the relationships, and for the 5 subjects, the averaged correlation coefficient *R* was 0.956±0.10 (mean±SD) for the regression between the SEMG RMS and the joint angle and 0.967±0.009 for that between the muscle deformation and the joint angle, respectively.

Table **1** shows the normalized SEMG RMS values measured for the 8 subjects in the ultrasound gel tests. The statistical analysis showed that the effect of ultrasound gel on the SEMG signals was not significant (*p* = 0.599 > 0.05).

## 4 DISCUSSION AND CONCLUSIONS

The results indicated that the system was capable of continuously capturing ultrasound, joint angle, and SEMG signals. A multithreaded approach used in the custom-developed program could realize the simultaneous acquisition of different signals in real-time. The temporal calibration experiments have been successfully performed for synchronizing the ultrasound image sequence, the SEMG signal, and the joint angle during the wrist extension. As the results reported in this paper are specific to our system, a similar calibration procedure would be required to validate other equivalent measurement systems. The cross-correlation algorithm used in the current method was able to extract the muscle deformation from the sequential ultrasound images. The tests of ultrasound gel area demonstrated that no significant effect of ultrasound gel on the SEMG signals could be observed.

The sampling rate achieved in the present system was relatively low (14 frame/s in the experiments), due to the limitations of the hardware settings. We found that the combined sampling rate was mainly limited by the CPU performance of the PC. In addition, due to the multithread protocol used in the system, the recorded SEMG signals showed some discontinuities between two adjacent frames, which limited the data points of the RMS and the frequency analysis to a single frame. This can be improved by interpolation algorithms for the SEMG RMS signals and selection of a high performance PC. It has also been observed that the gap between frames in the EMG signal could be significantly narrowed if the ultrasound images and EMG signals were not displayed on the PC screen during the data collection.

It was noted that the placement of ultrasound probe on the skin would introduce external motion artifacts to the SEMG measurements. In the experiments, the probe was not directly contacting the subjects’ skin and lots of gel was applied to fill the gap. The preliminary results were reported about the correlation among the muscle deformation, SEMG RMS signal, and joint angle in this paper. Further studies were being followed with more subjects. The synchronization and data collection technique presented in this study would help to investigate the correlation between the architectural and bioelectrical properties in not only static but also dynamic ways.

In conclusion, a new frame-synchronized SMG system for continuous acquisition and analysis of ultrasound images, SEMG signals and joint angles has been introduced in this paper. The experimental results demonstrated its good performance in data acquisition and analysis. Improvements in the system are required to increase the sampling rate and strengthen the data analysis. Based on its capability of capturing and analyzing synchronized ultrasound, EMG and other signals simultaneously, it is expected that the muscle activities can be investigated more comprehensively for different research and clinical applications.

## Figures and Tables

**Fig. (8):**
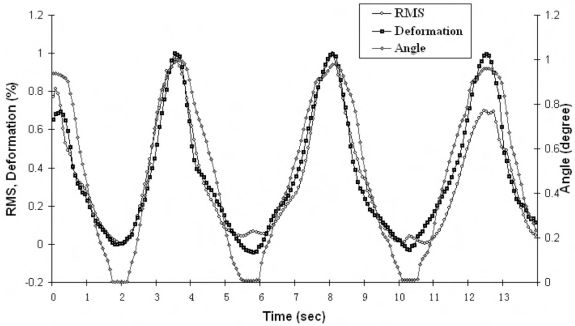
*In vivo* experimental results using the synchronized system.

**Fig. (9):**
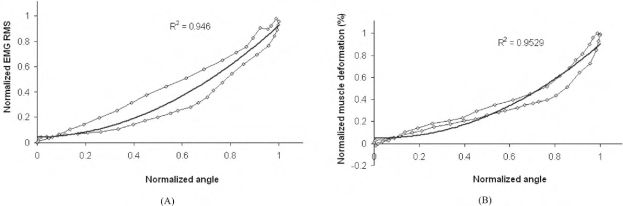
The typical results of the second-order polynomial regression of different signals. (**a**) The regression result between the wrist angles and the RMS amplitudes; and (**b**) the regression result between the wrist angles and muscle deformations.

**Table 1:** The Normalized RMS Amplitudes of SEMG Signals Collected from 8 Subjects in the Tests of Ultrasound Coupling Gel Area

Subject	RMS of SEMG			
	GA = 1×1 cm^2^	GA = 2×3 cm^2^	GA = 2×5 cm^2^	GA = 3×8 cm^2^
1	0.066	0.011	0.164	0.022
2	0.276	0.844	0.489	0.397
3	0.208	0.077	0.306	0.437
4	0.194	0.016	0.050	0.200
5	0.035	-0.001	-0.040	0.056
6	-0.236	-0.251	-0.199	-0.186
7	0.034	-0.018	-0.138	-0.144
8	-0.100	0.311	0.405	0.238
Mean	0.043	0.121	0.130	0.128
SD	0.175	0.330	0.254	0.231

GA: gel area.

## References

[1] Lieber RL, Fridén J (2000). “Functional and clinical significance of skeletal muscle architecture”. Muscle Nerve.

[2] McGill KC (2004). “Surface electromyogram signal modeling”. Med. Biol. Eng. Comput.

[3] Valls-Solé J, Montero J (2004). “Role of EMG evaluation in muscle hyperactivity syndromes”. J. Neurol.

[4] Walker FO, Cartwright MS, Wiesler ER, Caress J (2004). “Ultrasound of nerve and muscle”. Clin. Neurophysiol.

[5] Maganaris CN, Baltzopoulos V, Sargeant AJ (2006). “Human calf muscle responses during repeated isometric plantarflexions”. J. Biomechanics.

[6] Rutherford OM, Jones DA (1992). “Measurement of fibre pennation using ultrasound in the human quadriceps *in vivo*”. Eur. J. Appl. Physiol.

[7] Narici MV, Binzoni T, Hiltbrand E, Fasel J, Terrier F, Cerretelli P (1996). “*In vivo* human gastrocnemius architecture with changing joint angle at rest and during graded isometric contraction”. J. Physiol.

[8] Fukunaga T, Ichinose Y, Ito M, Kawakami Y, Fukashiro S (1997). “Determination of fascicle length and pennation in a contracting human muscle *in vivo*”. J. Appl. Physiol.

[9] Nordander C, Willner J, Hansson GA, Larsson B, Unge J, Granquist L, Skerfving S (2003). “Influence of the subcutaneous fat layer, as measured by ultrasound, skinfold calipers and BMI, on the EMG amplitude”. Eur. J. Appl. Physiol.

[10] Peschers UM, Gingelmaier A, Jundt K, Leib B, Dimpfl T (2001). “Evaluation of pelvic floor muscle strength using four different techniques”. Int. Urogynecol. J.

[11] Hodges PW, Pengel LHM, Herbert RD, Gandevia SC (2003). “Measurement of muscle contraction with ultrasound imaging”. Muscle Nerve.

[12] McMeeken JM, Beith ID, Newham DJ, Milligan P, Critchley DJ (2004). “The relationship between EMG and Change in thickness of transversus abdominis”. Clin. Biomech.

[13] Ferreira PH, Ferreira ML, Hodges PW (2004). “Changes in recruitment of the abdominal muscles in people with low back pain Ultrasound measurement of muscle activity”. Spine.

[14] Mademli L, Arampatzis A (2005). “Behaviour of the human gastrocnemius muscle architecture during submaximal isometric fatigue”. Euro. J. Appl. Physiol.

[15] Mademli L, Arampatzis A, Walsh M (2006). “Effect of muscle fatigue on the compliance of the gastrocnemius medialis tendon and aponeurosis”. J. Biomechanics.

[16] Shi J, Zheng YP, Chen X, Huang QH (2007). “Assessment of muscle fatigue using sonomyography: muscle thickness change detected from ultrasound images”. Med. Eng. Phys.

[17] Zheng YP, Chan MMF, Shi J, Chen X, Huang QH (2006). “Sonomyography: monitoring morphological changes of forearm muscles in actions with the feasibility for the control of powered prosthesis”. Med. Eng. Phy.

[18] Huang QH, Zheng YP, Lu MH, Chi ZR (2005). “Development of a portable 3D ultrasound imaging system for musculoskeletal tissues”. Ultrasonics.

